# Intestinal parasite infections in a rural community of Rio de Janeiro (Brazil): Prevalence and genetic diversity of *Blastocystis* subtypes

**DOI:** 10.1371/journal.pone.0193860

**Published:** 2018-03-09

**Authors:** Carolina Valença Barbosa, Magali Muniz Barreto, Rosemary de Jesus Andrade, Fernando Sodré, Claudia Masini d’Avila-Levy, José Mauro Peralta, Ricardo Pereira Igreja, Heloisa Werneck de Macedo, Helena Lucia Carneiro Santos

**Affiliations:** 1 Laboratório de Estudos Integrados em Protozoologia, Instituto Oswaldo Cruz/Fundação Oswaldo Cruz (FIOCRUZ), Rio de Janeiro, Brazil; 2 Laboratório de Avaliação e Promoção da Saúde Ambiental, Instituto Oswaldo Cruz/FIOCRUZ, Rio de Janeiro, Brazil; 3 Departamento de Ginecologia e Obstetrícia, Instituto Fernandes Figueira/FIOCRUZ, Rio de Janeiro, Brazil; 4 Laboratório de Parasitologia, Departamento de Patologia, Hospital Universitário Antônio Pedro, Universidade Federal Fluminense, Rio de Janeiro, Brazil; 5 Instituto de Microbiologia Paulo de Goes, UFRJ, Rio de Janeiro, Brazil; 6 Departamento de Medicina Preventiva da Faculdade de Medicina, Universidade Federal do Rio de Janeiro (UFRJ), Rio de Janeiro, Brazil; Consejo Nacional de Investigaciones Cientificas y Tecnicas, Fundación Mundo Sano, ARGENTINA

## Abstract

**Background:**

Intestinal parasitic infections are considered a serious public health problem and widely distributed worldwide, mainly in urban and rural environments of tropical and subtropical countries. Globally, soil-transmitted helminths and protozoa are the most common intestinal parasites. *Blastocystis* sp. is a highly prevalent suspected pathogenic protozoan, and considered an unusual protist due to its significant genetic diversity and host plasticity.

**Methodology/main findings:**

A total of 294 stool samples were collected from inhabitants of three rural valleys in Rio de Janeiro, Brazil. The stool samples were evaluated by parasitological methods, fecal culture, nested PCR and PCR/Sequencing. Overall prevalence by parasitological analyses was 64.3% (189 out of 294 cases). *Blastocystis* sp. (55.8%) was the most prevalent, followed by *Endolimax nana* (18.7%), *Entamoeba histolytica* complex (7.1%), hookworm infection (7.1%), *Entomoeba coli* (5.8%), *Giardia intestinalis* (4.1%), *Iodamoeba butchilii* (1.0%), *Trichuris trichiura* (1.0%), *Pentatrichomonas hominis* (0.7%), *Enterobius vermicularis* (0.7%), *Ascaris lumbricoides* (0.7%) and *Strongyloides stercoralis* (0.7%). Prevalence of IPIs was significantly different by gender. Phylogenetic analysis of *Blastocystis* sp. and BLAST search revealed five different subtypes: ST3 (34.0%), ST1 (27.0%), ST2 (27.0%), ST4 (3.5%), ST8 (7.0%) and a non-identified subtype.

**Conclusions/significance:**

Our findings demonstrate that intestinal parasite infection rates in rural areas of the Sumidouro municipality of Rio de Janeiro, Brazil are still high and remain a challenge to public health. Moreover, our data reveals significant genetic heterogeneity of *Blastocystis* sp. subtypes and a possible novel subtype, whose confirmation will require additional data. Our study contributes to the understanding of potential routes of transmission, epidemiology, and genetic diversity of *Blastocystis* sp. in rural areas both at a regional and global scale.

## Introduction

Intestinal parasitic infections (IPIs) are ubiquitous in humans, both in urban and rural environments from tropical and subtropical countries. The poorest and most deprived communities are at increased risk of intestinal parasitic infections, which are present in more than a quarter of the world’s population [1–3]. The frequency of intestinal parasites is an indicator of low socioeconomic development of a population, which is directly associated with educational deficits and poor sanitary conditions [4]. However, in tropical developing countries, rural life is by itself associated with a high risk of infections due to negligible health knowledge, lower socio-economic conditions, inadequate environmental sanitation, insufficient water supply [5, 6] and higher contact rates with wildlife and domestic reservoirs of infection [7–9]. Rural populations experience a vicious cycle of malnutrition and re-infections leading to continuous morbidity and perpetuation of poverty cycles. IPIs are a public health problem caused by helminths and intestinal protozoa [3, 10, 11]. Globally, the soil-transmitted helminthes *Ascaris lumbricoides*, *hookworm* and *Trichuris trichiura* and the protozoan *Entamoeba histolytica*, *Giardia intestinalis* and *Cryptosporidium* sp. are the most common intestinal parasites [12]. Except for *E*. *histolytica*, *Cryptosporidium spp* and *Balantidium coli*, they are unable to invade the mucosal tissues or other organs. In the recent years, there has been a growing recognition that *Blastocystis* sp. presents pathogenic potential, although its virulence mechanisms are not understood. They infect a wide range of animals including birds, amphibians, reptiles, insects and mammals, including humans [13–18]. Although the current knowledge of reservoirs for human infection is limited, it is known that a vehicle for *Blastocystis* sp. transmission is close contact with animals [15, 16, 19]. *Blastocystis* sp. has potential pandemic distribution, possibly reaching 30% in industrialized countries and up to 76% in developing nations [12, 20, 21].

Analysis of the small-subunit ribosomal RNA of *Blastocystis* sp. isolates revealed substantial genetic diversity, represented by 17 genetically distinct ribosomal lineages (subtypes/ST1-ST17) [18, 22]. Ten subtypes (ST1-ST9, and ST12) have been isolated from human and animal fecal samples (except ST9) [7, 16, 23–25], whereas others were exclusively found in non-human hosts [14, 26]. The significance of *Blastocystis* sp. to public health, potential zoonotic differences and clinical outcomes may be related to specific subtype, a hypothesis that has been the topic of recent debate and requires further examination [15, 27].

The molecular epidemiology of *Blastocystis* sp. infections is still unknown in many parts of the world. Brazil is a continental country and information on the distribution of STs in the country is still incipient, but a high prevalence of *Blastocystis* sp. has been reported both in urban and rural environments. Moreover, IPIs are still a major public health problem, especially among the impoverished and underprivileged communities living in rural and remote areas, which represent an important target of public health interventions. To address this gap, we investigated the prevalence of intestinal parasites and distribution of *Blastocystis* sp. STs in inhabitants of rural areas of Rio de Janeiro, Brazil. We report a high prevalence of intestinal parasitic infections and a wide range of *Blastocystis* sp. STs in the area.

## Materials and methods

### Study population

A cross-sectional study was carried out from October to December of 2013 in Pamparrão, Porteira Verde and Porteira Verde Alta valleys, in rural area of Sumidouro, State of Rio de Janeiro, Brazil (22° 02’ 46” S; 42° 41’ 21”W). The valleys are populated by small rural properties characterized by vegetable crops and pasture. Total population is around 14,900 inhabitants, and 87.7% of people work in agriculture [28]. The Sumidouro municipality is located in the central mountains bordering the Serra do Mar in Rio de Janeiro, at altitudes varying between 264 and 1,300 meters. The region exhibits marked climatic contrasts due to its peculiar geographical position and humid-mesothermic climate [29], with average rainfall between 153.5 mm and 269.4 mm.

Participants were informed about the study aims, potential risks and benefits, and provided written informed consent followed by signatures of two external witnesses. Relatives or legal tutors provided informed consent for children. Participation was voluntary and people could withdraw from the study at any time without further obligation. To preserve anonymity, each study participant was given a unique identification number. At the end of the study, all participants diagnosed with enteric parasitic pathogens received proper therapy. Treatment was not offered to individuals infected with *Blastocystis* sp., because its pathogenic potential is still a topic of debate. The study protocol was reviewed and approved by the Human Ethics Committee of the Faculdade Medicina from Universidade Federal Fluminense (CAAE 35028314.2.0000.5 243).

### Parasitological assays

A total of 294 unpreserved stool samples were collected from participants at home, kept at 4°C and transported to the laboratory at the Fundação Oswaldo Cruz in ice packs. Fresh unpreserved stool samples were fractionated into two aliquots upon receipt. Parasitological examination was performed by spontaneous sedimentation technique (also known as Lutz technique or Hoffman, Pons and Janer technique)[30] and flotation in saturated sodium chloride solution density 1.2 g/mL[31]. Direct *in vitro* xenic cultivation of *Blastocystis* sp. from an aliquot of stool samples was performed using Pavlova’s medium, supplemented with 10% heat inactivated adult bovine serum and penicillin-streptomycin antibiotics and incubated at 37°C [32]. Screening of *Blastocystis* sp. cultures was performed using standard light microscopy during the first three days of incubation. When typical forms of the parasite (vacuolar, granular and amoeboid forms) were observed, *Blastocystis* sp. culture aliquots were frozen at -20°C for subsequent DNA extraction. An aliquot of fresh unpreserved stool samples was stored at -20°C prior to analysis. Statistical analyses were carried out with Epi-Info 3.5.1. Relationships between variables were examined by chi-square tests with significance levels set at 5%.

### Molecular analysis

DNA extraction from cultured samples, positive for *Blastocystis* sp. and from human stools, positive for E. *histolytica* complex was carried out using the the Qiamp DNA Stool Mini Kit (Qiagen, Valencia, CA) according to manufacturer’s recommendations. Samples exhibiting structures of the *E*. *histolytica* complex under microscopic examination were subjected to DNA amplification.

Nested PCR targeting 18S-like ribosomal RNA gene was applied to genetically identify the *E*. *histolytica*, *E*. *dispar* and *E*. *moshkovskii* complex [33]. First-round PCR for detection of *Entamoeba* genus used the forward primer E-1 (5' TAAGATGCACGAG AGCGAAA3') and reverse primer E-2 (5' GTACAAAG GGCAGGGACGTA 3'). PCR reaction was performed in a 50 μL volume, with the final mix containing 10× PCR buffer, 1.25 mM of dNTPs, 2.5 mM MgCl_2_, 10 pmoles of each primer, 2.5 U of Taq polymerase (Invitrogen Life Technologies, Carlsbad, CA, USA), and 2.5 μlL of DNA template. PCR cycles consisted of 5 min at 95°C and 40 cycles of 30 s at 95°C, 60 s at 56°C, and 60 s at 72°C, with a final step of 2 min at 72°C. Afterwards, the primary PCR products were subjected to second-round PCRs for Entamoeba species-specific characterization, which had multiple primers sets in the same tube. Amplification was achieved using the following primer sets EH-1 (5’-AAGCATTGTTTCTAGA TCTGAG-3’) and EH-2 (5’-AAGAGGTCTAACCGAAATTAG-3’) to detect *E*. *histolytica* (439 bp); ED-1 (5’-TCTAATTTCGATTAGAACTCT-3’) and ED-2 (5’-TCCCTACCTATTAGACATAGC-3’) to detect *E*. *dispar* (174 bp); Mos-1 (5’-GAAACCAAGAGTTTCACAAC-3’) and Mos-2 (5’-CAATATAAGGCTTGGATGA T-3’) to detect *E*. *moshkovskii* (553 bp) [33]. For the second PCR round, only the annealing temperature was changed to 48°C, while all other amplification cycle parameters were unchanged. Amplified products were visualized with stained with Gelred (Biotium Inc., Hayward, CA, USA) under UV transllumination after electrophoresis on 1.5% agarose gels. PCR amplification of partial SSU rDNA to identify subtypes of *Blastocystis* sp. was performed as protocol previously described [17], which amplifies a fragment approximately 500 bp long.

Genomic DNA was subjected to PCR analysis using primer pair Blast 505–532 *(*5’ GGA GGT AGT GAC AAT AAATC 3’*)* and Blast 998–1017 *(*5’TGC TTT CGC ACT TGT TCATC 3’*)*. The PCR reaction was performed in a final volume of 50 μL and each reaction contained 100 mM of TrisHCl (pH 9.0), 500 mM of KCl, 1.5 mM of MgCl_2_, 200 μM of dATP, dGTP, dCTP, and dTTP each, 0.2 μM of primer, 1.5 U of Taq DNA polymerase (Invitrogen Life Technologies, Carlsbad, CA, USA), 0.05% of bovine serum albumin (BSA), and 5 μL of DNA sample. PCR conditions consisted of an initial cycle at 95°C for 5 min, 35 cycles including denaturing at 95°C for 30 s, annealing at 55°C for 30 s, extending at 72°C for 2 min, with a final step of 2 min at 72°C. PCR products were electrophoresed in 1.5% agarose gel in Tris-borate ethylenediamine tetraacetic acid (EDTA) buffer, stained with Gelred (Biotium Inc., Hayward, CA, USA) and visualized under UV transllumination. Amplicons were purified using the Wizard^®^ SV gel and PCR clean up system kit (Promega, Madison, WI, USA) and sequenced in both directions using Big Dye chemistries in an ABI3100 sequencer analyzer (Applied Biosystems, Foster City, CA). Our SSU rDNA partial gene sequences were deposited in GenBank under accession numbers KX523972 to KX524055. They were compared to available GenBank sequences using the BLASTN program on the National Center for Biotechnology Information (NCBI) server (http://www.ncbi.nlm.nih.gov/BLAST). In addition, DNA sequences of ST1 to ST9 of *Blastocystis* sp. were downloaded from Genbank, and multiple sequence alignment was performed using the ClustalW algorithm of the MEGA software version 6.0 [34].

### Phylogenetic analysis

To identify the phylogenetic position of isolates, phylogenetic trees were created using *Proteromonas lacertae*as, as the outgroup [35, 36]. Phylogenetic trees were constructed using two probabilistic methods: Maximum Likelihood (ML) and Bayesian Inference (BI), which are based on HKY + G (gamma distribution of rates with four rate categories) model [37]. The best model of nucleotide substitutions was selected based on the Akaike Information Criterion using Jmodeltest [38] for ML, and Mrmodeltest for BI [39]. The ML tree was created using PhyML 3.1 [40] and BI in MrBayes software version 3.2 [41]. Bootstrap values were calculated through the analysis of 1000 replicates for the ML analysis and through Bayesian posterior probability analysis using the MCMC algorithm, with four chains. Chains were run for 10^7^ generations and sampled every 100 generations. Convergence was assessed by evaluating the average standard deviation of split frequencies, which were well below the recommended values (< 0.01). The first 25% of the sampled trees were discarded as burn-in for each data set, and the consensus tree topology and nodal support were estimated from the remaining samples as posterior probability values.

## Results

A total of 294 individuals participated in this study, 145 (49.3%) males and 149 (50.7%) females (age range = 2–87 years, mean age = 38.3 years). Participants were stratified by age and gender (Table 1). There were 21 (7.1%) participants aged 1–5 years, 24 (8.2%) aged 6–14 years, 190 (64.6%) aged 15–55 years and 59 (20.1%) participants aged 55 years and over. A total of 126 individuals (42.8%) provided three stool samples, 99 (33.6%) two samples and 69 (23.4%) one sample. The study population was characterized predominantly by adults (81.2%). Overall, 189 of participants out of 294 (64.3%) were infected with at least one enteric parasite, with gender specific prevalence being 54.8% in males and 45.2% females. Prevalence was significantly different between the genders (χ^2^ = 6.82; p = 0.009). The total prevalence of protozoan infections was 60.2%. The most predominant species was *Blastocystis* sp., followed by *Endolimax nana*, *E*. *histolytica* complex, *Entamoeba coli*, and *Giardia intestinalis*. The total prevalence of helminth infections was 13.7%. Hookworm was the most prevalent helminth specie, followed by *Trichuris trichiura* (Table 1).

**Table 1:** Distribution of intestinal parasites according to age groups and gender.

	Age group, in years				Gender*		
	1–5	6–14	15–55	55 y plus	M	F	Total (%)
**N° (%)**	21(7.1)	24(8.2)	190(64.6)	59(20.1)	145(49.3)	149(50.7)	294(100)
**Pos**	8 (38.0)	12 (50.0)	128 (67.3)	41 (69.5)	103 (71.0)	86 (57.7)	189 (64.3)
**Neg**	13 (62.0)	12 (50.0	62 (32.7)	18 (30.5)	42 (29.0)	63 (42.3)	105 (35.7)
**Blast**	7 (33.3)	10 (41.7)	110 (57.9)	37 62.7)	92 (63.4)	72 (48.3)	164 (55.8)
**Ec**	2 (9.5)	1 (4.2)	9 (4.7)	5 (8.5)	10 (6.9)	7 (4.7)	17 (5.8)
**Eh complex**	2 (9.5)	1 (4.2)	14 (7.4)	4 (6.8)	12 (8.3)	9 (6.0)	21 (7.1)
**En**	2 (9.5)	0 (0.0)	44 (23.2)	9 (15.3)	33 (22.8)	22 (14.8)	55 (18.7)
**Gi**	2 (9.5)	3 (12.5)	5 (2.6)	2 (3.4)	9 (6.2)	3 (2.0)	12 (4.1)
**Ib**	0 (0.0)	0 (0.0)	1 (0.5)	2 (3.4)	2 (1.4)	1 (0.7)	3 (1.0)
**Hook**	0 (0.0)	0 (0.0)	19 (10.0)	2 (3.4)	13 (9.0)	8 (5.4)	21 (7.1)
**Ss**	0 (0.0)	0 (0.0)	1 (0.5)	0 (0.0)	1 (0.7)	0 (0.0)	1 (0.3)
**Ph**	0 (0.0)	1 (4.2)	1 (0.5)	0 (0.0)	0 (0.0)	2 (1.3)	2 (0.7)
**Al**	1 (4.8)	0 (0.0)	0 (0.0)	1 (1.7)	1 (0.7	1 (0.7)	2 (0.7)
**Tt**	0 (0.0)	0 (0.0)	3 (1.6)	0 (0.0)	1 (0.7)	2 (1.3)	3 (1.0)
**Ev**	0 (0.0)	0 (0.0)	0 (0.0)	2 (3.4)	1 (0.7)	1 (0.7)	2 (0.7)

Pos = positive samples; neg = negative samples; Blast = *Blastocystis* sp; Ec = *Entamoeba coli*; En = *Endolimax nana*; Eh complex = *E*. *histolytica*, *E*. *dispar*, *or E*. *moshkovskii* Gi = *Giardia intestinalis*; Ib = *Iodamoeba butschlii*; Hook = hookworm; Ss: *Strongyloides stercoralis;* Tt = *Trichiuris trichiura*; Ph = *Pentatrichomonas hominis*; Al = *Ascaris lumbricoides*; EV = *Enterobius vermicularis*. F = female; M = male; y = years.

*(p = 0.009)

**Fig 1:**
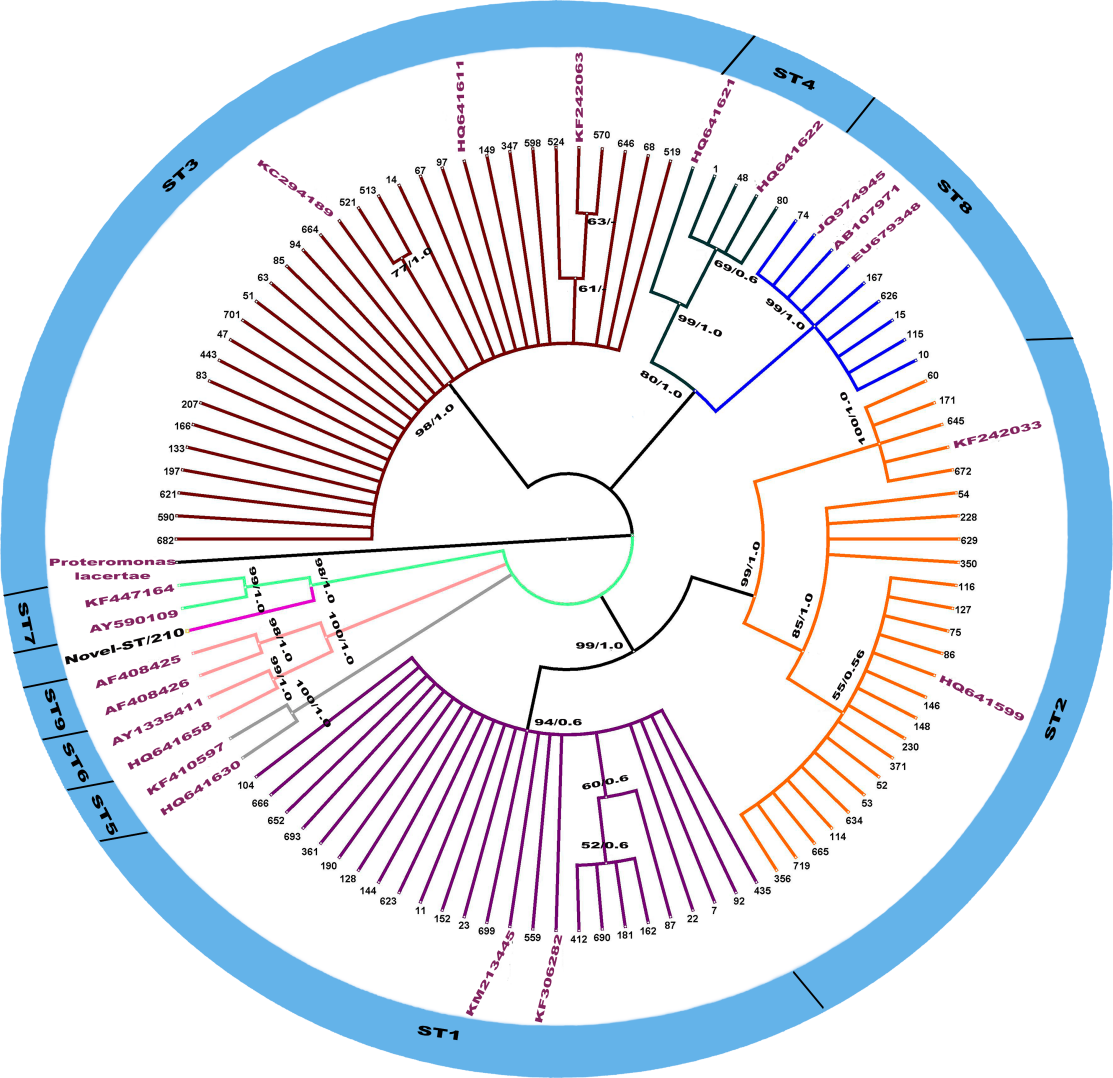
Maximum likelihood (ML) and Bayesian inference (BI) trees based on the partial sequences of the partial SSU rDNA (437 bp) of *Blastocystis* sp. isolates of the present study. The high genetic divergence of rDNA gene revealed six clusters (denoted by ST1, 2, 3, 4, 8 and Novel-ST). The numbers along branches correspond to bootstrap values and Bayesian posterior probability values (values above 50% are shown) in the order: MP/BI.

All 21 microscopically samples positive for *E*. *histolytica* complex were successfully amplified by nested PCR. Of those samples, 20 (95%) were positive for *E*. *dispar* and one (5.0%) had a mixed infection of *E*. *histolytica* and *E*. *dispar*. Culture and Hoffman techniques, both used for diagnosis of *Blastocystis* sp., exhibited very similar sensibility: 118 of 164 (71.9%) samples were diagnosed as positive by both techniques, 24 only by the use of culture and 22 only by the Hoffman technique. There was no statistical difference between techniques (p = 0.888). Of the 164 samples positive for *Blastocystis* sp., a subset of 85 (51.8%) was successfully sequenced. The sequencing of partial SSU rDNA gene indicated the presence of subtypes: ST3 (34.0%), ST1 (27.0%), ST2 (27.0%), ST4 (3.5%), ST8 (7.0%) and a non-identified subtype (ID 210). Sequences showed high identity (98–100%) to sequences available at the NCBI database, while sample ID 210 returned a BLAST identity of 93%. Topology of the constructed phylogenetic tree revealed that ST1, ST2, ST3, ST4, ST8 and the non-identified ST clustered into well-supported clades (with grouped bootstrap values over 70.0% and Bayesian posterior probabilities) (Fig 1). The non-identified *Blastocystis* sp. ST is evolutionarily closer to ST7. Intra-ST diversity was observed in ST1, ST2 and ST3 as indicated by their small clades.

## Discussion

We assessed the prevalence of IPIs and identity of *Blastocystis* sp. subtypes in a rural community of Rio de Janeiro, Brazil. IPIs have been described as the greatest worldwide cause of diseases [42]. Current epidemiological evaluations suggest that 450 million people, especially in developing countries, may host multiple parasite species [1]. Our results showed that 64.3% of participants presented IPIs, with the most common human parasite being *Blastocystis* sp., in accordance to previous work across Brazilian regions [12, 43–46]. Although IPIs had received attention in Sumidouro as early as 1995, most of these efforts targeted Schistosomiasis. Gonçalves et al. [47] previously reported a prevalence of 13% of hookworm, 5% of *Ascaris lumbricoides*, 20% of *Entamoeba coli* and 10% of *Giardia intestinalis* in the Sumidouro population. The prevalence of protist parasites that we are reporting is much higher than of helminths, which may be explained by self-medication (antihelminths) practiced by the population, or by the use of pesticides in agriculture above recommended doses, which may have a significant effect on soil-transmitted helminths survival. However, these hypotheses still require investigation.

We also identified statistically significant differences in infection rates between men and women. Several investigations have reported a higher prevalence of parasitic infection in males than females [48, 49]. A possible explanation is the higher engagement of males in farming activities. Most of the Sumidouro population is rural and their most important economic activities are cattle breeding and vegetable cultivation. People from rural communities usually eat fresh food, drink untreated water and disposal of sewage is generally inadequate [50], resulting in increased infection risk. The diversity of protist parasites reveled by our analyses suggest that their distribution depends on factors such as environmental conditions, lack of sanitation and health education, confirming previous studies in rural communities [51, 52]. It was shown that short-term xenic *in vitro* culture (XIVC) is a more sensitive diagnostic method than parasitological examination [53]. Contrary to the expectations, our findings showed that the proportion of specimens negative by parasitological examination but positive by xenic *in vitro* culture, and vice-versa, were similar. The likely explanation is that the fecal samples did not contain viable *Blastocystis* sp. or alternatively cultured organisms were scant and imperceptible thereby avoiding detection by microscopy. Moreover, the screening of *Blastocystis* sp. cultures was performed in the first three days of incubation, when parasite count was low due to slow growth rates. Screening after a week of cultivation for example might reveal additional positives missed by the analyses performed herein.

The predominant *Blastocystis* sp. subtype was ST3, ST1 and ST2. STs 1, 2 and 3 had already been identified as the most abundant subtypes in previous studies [53–56]. Recently, Ramirez et al. [21] have reported percentages ranging from 15 to 92% regarding these subtypes. Other investigations have singled out ST3 as the most frequent subtype [18, 57–64]. Subtypes 1 and 2 have been identified both in humans and a wide range of synanthropic animals such as pigs, rats, dogs, horses, monkeys, cattle and chickens [15, 16, 65]. A recent study using animal models revealed that various human *Blastocystis* sp. subtypes, including ST1 and ST2, could infect chickens and rats [66, 67], suggesting the zoonotic potential of human *Blastocystis* sp. isolates. We also observed the occurrence of ST4 in only three residents, although this subtype is the second most common in the UK and commonly found across Europe [15, 27, 68–70]. In Colombia, ST4 was identified in a few humans and non-human primates [21]. Rodents were also shown to be reservoir hosts of ST4 [23, 71]. Brazil was the only country in South America where ST8 has been identified [21], although it was also found in Denmark, the UK, Italy and Australia [62, 65, 72, 73]. Large American opossums and non-human primates can be infected by ST8, which is rarely found in other hosts [18]. We could not identify the subtype from sample 210. However, our phylogenetic tree shows that the sample is closely related to ST7. A similar case was reported by Ramirez et al. [21] who reported a novel ST closely related to ST1 and found in six South American countries including Brazil. Further analyses and full-length sequencing of the SSU-rDNA gene are required to truly confirm the existence of the novel ST.

## Conclusion

Our study revealed the presence of IPIs in a rural community in Brazil, with high prevalence and a wide diversity of *Blastocystis* sp. subtypes, and possibly a new subtype of the protozoan. Our findings reinforce the need for additional studies of *Blastocystis* sp. subtype diversity, which may contribute to more efficient identification of infection sources and potential transmission routes under various ecological conditions, as well as a re-evaluation of control strategies by public health services.

## Supporting information

S1 Table New sequences generated as a result of this study. (DOCX)
