# Supplementary material for: Intestinal parasite infections in a rural community of Rio de Janeiro (Brazil): Prevalence and genetic diversity of Blastocystis subtypes
Source: PLoS One. 2018 Mar 9;13(3):e0193860. doi: 10.1371/journal.pone.0193860 (PMC5844535; doi:10.1371/journal.pone.0193860)
Supplement: S1 Table — (DOCX) [file pone.0193860.s001.docx]

Table S1. New sequences generated as a resulto of this study

| Isolates | *Blastocystis* ST | Genbank Acession number |
| --- | --- | --- |
| 210 | Novel-ST | KX523972 |
| 7 | ST1 | KX523985 |
| 11 | ST1 | KX524052 |
| 22 | ST1 | KX524030 |
| 23 | ST1 | KX524028 |
| 87 | ST1 | KX523976 |
| 92 | ST1 | KX523975 |
| 104 | ST1 | KX524053 |
| 128 | ST1 | KX524047 |
| 144 | ST1 | KX524044 |
| 152 | ST1 | KX524039 |
| 162 | ST1 | KX524038 |
| 181 | ST1 | KX524034 |
| 190 | ST1 | KX524033 |
| 361 | ST1 | KX524023 |
| 412 | ST1 | KX524021 |
| 435 | ST1 | KX524020 |
| 559 | ST1 | KX524008 |
| 623 | ST1 | KX524003 |
| 652 | ST1 | KX523995 |
| 666 | ST1 | KX523993 |
| 690 | ST1 | KX523988 |
| 693 | ST1 | KX523987 |
| 699 | ST1 | KX523986 |
| 52 | ST2 | KX524013 |
| 53 | ST2 | KX524010 |
| 54 | ST2 | KX524009 |
| 60 | ST2 | KX524005 |
| 75 | ST2 | KX523981 |
| 86 | ST2 | KX523977 |
| 114 | ST2 | KX524051 |
| 116 | ST2 | KX524049 |
| 127 | ST2 | KX524048 |
| 146 | ST2 | KX524043 |
| 148 | ST2 | KX524042 |
| 171 | ST2 | KX524035 |
| 228 | ST2 | KX524029 |
| 230 | ST2 | KX524027 |
| 350 | ST2 | KX524025 |
| 356 | ST2 | KX524024 |
| 371 | ST2 | KX524022 |
| 629 | ST2 | KX524001 |
| 634 | ST2 | KX523999 |
| 645 | ST2 | KX523998 |
| 665 | ST2 | KX523994 |
| 672 | ST2 | KX523991 |
| 719 | ST2 | KX523983 |
| 14 | ST3 | KX524045 |
| 47 | ST3 | KX524018 |
| 51 | ST3 | KX524016 |
| 63 | ST3 | KX524000 |
| 67 | ST3 | KX523992 |
| 68 | ST3 | KX523990 |
| 83 | ST3 | KX523979 |
| 85 | ST3 | KX523978 |
| 94 | ST3 | KX523974 |
| 97 | ST3 | KX523973 |
| 133 | ST3 | KX524046 |
| 149 | ST3 | KX524041 |
| 166 | ST3 | KX524037 |
| 197 | ST3 | KX524032 |
| 207 | ST3 | KX524031 |
| 347 | ST3 | KX524026 |
| 443 | ST3 | KX524019 |
| 513 | ST3 | KX524015 |
| 519 | ST3 | KX524014 |
| 521 | ST3 | KX524012 |
| 524 | ST3 | KX524011 |
| 570 | ST3 | KX524007 |
| 590 | ST3 | KX524006 |
| 621 | ST3 | KX524004 |
| 646 | ST3 | KX523997 |
| 664 | ST3 | KX523996 |
| 682 | ST3 | KX523989 |
| 701 | ST3 | KX523984 |
| 598 | ST3 | MG309719 |
| 1 | ST4 | KX524055 |
| 48 | ST4 | KX524017 |
| 80 | ST4 | KX523980 |
| 10 | ST8 | KX524054 |
| 15 | ST8 | KX524040 |
| 74 | ST8 | KX523982 |
| 115 | ST8 | KX524050 |
| 167 | ST8 | KX524036 |
| 626 | ST8 | KX524002 |
